# Supplementary material for: Analyses of allele age and fitness impact reveal human beneficial alleles to be older than neutral controls
Source: bioRxiv. 2023 Oct 11:2023.10.09.561569. Preprint. [Version 2] doi: 10.1101/2023.10.09.561569 (PMC10592680; doi:10.1101/2023.10.09.561569)

Supplementary information

For "Balancing selection is common for beneficial alleles in a human population" by Pivirotto et al

Supplementary Table 1. Results of simulation-based tests of dispersion of positive ΔEP SNPs. 2

Supplemental Table 2. Gene ontology results 3

Supplemental Table 3. F_{ST} Values across ΔEP spectrum of values. 6

Supplementary Table 4. ΔEP measures for fixed and polymorphic alleles.. 7

Supplementary Table 5. Statistical power for detecting excess heterozygosity 8

Supplementary Figure 1. Distributions of derived polymorphism frequency in UK10K. 9

Supplementary Table 1. Results of simulation-based tests of dispersion of positive ΔEP SNPs.

Chromosome	# Genes	Observed Mean SNP Density/bp	Mean simulated density	Observed Variance in Density	Mean simulated variance in density	Estimated probability of simulating variance higher than observed
1	1849	0.00035	0.00034	1.21E-06	3.50E-07	0
2	1134	0.0003	0.0003	1.45E-06	2.72E-07	0
3	1000	0.00027	0.00027	8.35E-07	2.39E-07	0
4	695	0.00039	0.00039	1.70E-06	3.75E-07	0
5	815	0.00043	0.00043	2.49E-06	4.10E-07	0
6	950	0.00026	0.00025	6.85E-07	2.69E-07	0
7	807	0.00038	0.00038	1.75E-06	3.65E-07	0
8	603	0.00038	0.00039	1.93E-06	3.70E-07	0
9	701	0.0004	0.0004	1.67E-06	3.81E-07	0
10	667	0.00035	0.00036	1.67E-06	3.25E-07	0
11	1207	0.00055	0.00055	2.87E-06	5.76E-07	0
12	965	0.00025	0.00026	6.31E-07	2.47E-07	0
13	299	0.00023	0.00023	5.37E-07	1.95E-07	0
14	551	0.00023	0.00024	4.00E-07	2.27E-07	0
15	517	0.00021	0.00022	3.47E-07	1.83E-07	0
16	744	0.00057	0.00057	4.37E-06	5.82E-07	0
17	1052	0.00043	0.00043	1.80E-06	4.31E-07	0
18	251	0.00023	0.00023	3.11E-07	1.99E-07	0.055
19	1296	0.00076	0.00076	4.32E-06	7.38E-07	0
20	506	0.00035	0.00035	9.00E-07	3.99E-07	0
21	205	0.00064	0.00065	5.66E-06	9.64E-07	0
22	395	0.00049	0.00049	1.74E-06	4.78E-07	0

Supplemental Table 2. Gene ontology results

Panther Pathways	Observed	Expected	Fold Enrichment	Raw P Value	FDR
Plasminogen activating cascade	4	0.06	64.59	9.14E-07	7.31E-05
Blood coagulation	6	0.17	35.81	3.33E-08	5.32E-06

GO Biological Process	Observed	Expected	Fold Enrichment	Raw P Value	FDR
plasminogen activation	3	0.04	74.87	1.62E-05	1.82E-02
blood coagulation, fibrin clot formation	5	0.09	57.19	6.10E-08	3.19E-04
protein activation cascade	5	0.09	52.79	8.68E-08	3.40E-04
blood coagulation	7	0.63	11.11	4.08E-06	7.11E-03
coagulation	8	0.64	12.55	3.30E-07	1.04E-03
hemostasis	7	0.65	10.80	4.89E-06	6.97E-03
positive regulation of heterotypic cell-cell adhesion	3	0.05	54.90	3.60E-05	3.32E-02
acute-phase response	6	0.15	39.22	2.03E-08	3.18E-04
acute inflammatory response	7	0.3	23.43	3.39E-08	2.65E-04
platelet aggregation	4	0.16	25.54	2.54E-05	2.65E-02
cell-cell adhesion	10	1.98	5.06	3.02E-05	2.96E-02
negative regulation of endopeptidase activity	8	0.9	8.86	4.10E-06	6.43E-03
regulation of endopeptidase activity	10	1.54	6.51	3.54E-06	6.95E-03
regulation of peptidase activity	11	1.65	6.68	8.55E-07	1.91E-03
regulation of proteolysis	14	2.71	5.17	4.91E-07	1.28E-03
negative regulation of peptidase activity	8	0.94	8.55	5.29E-06	6.91E-03
negative regulation of proteolysis	9	1.26	7.16	5.33E-06	6.43E-03

GO Molecular Function	Observed	Expected	Fold Enrichment	Raw P Value	FDR
serine-type endopeptidase inhibitor activity	6	0.37	16.31	2.52E-06	2.51E-03
endopeptidase inhibitor activity	8	0.67	12.00	4.57E-07	1.14E-03
endopeptidase regulator activity	8	0.72	11.15	7.81E-07	9.72E-04
peptidase regulator activity	9	0.86	10.51	2.46E-07	1.22E-03
peptidase inhibitor activity	8	0.69	11.56	6.01E-07	9.96E-04
protease binding	6	0.51	11.68	1.57E-05	1.30E-02

GO Cellular Component	Observed	Expected	Fold Enrichment	Raw P Value	FDR
fibrinogen complex	4	0.03	> 100	7.75E-08	1.98E-05
extracellular space	26	12.49	2.08	1.41E-04	1.92E-02
extracellular region	30	16.01	1.87	3.16E-04	3.23E-02
endocytic vesicle lumen	3	0.08	37.43	9.97E-05	1.57E-02
platelet alpha granule lumen	7	0.24	28.68	9.23E-09	3.77E-06
platelet alpha granule	7	0.33	21.12	6.62E-08	1.93E-05
secretory granule	13	3.2	4.06	1.75E-05	2.98E-03
secretory vesicle	13	3.83	3.40	1.07E-04	1.57E-02
secretory granule lumen	12	1.17	10.29	2.56E-09	1.74E-06
cytoplasmic vesicle lumen	12	1.18	10.20	2.83E-09	1.45E-06
vesicle lumen	13	1.18	10.98	2.43E-10	2.48E-07
blood microparticle	13	0.52	24.78	1.43E-14	2.93E-11
endoplasmic reticulum lumen	11	1.14	9.62	2.46E-08	8.38E-06
collagen-containing extracellular matrix	11	1.58	6.96	5.79E-07	1.31E-04
extracellular matrix	11	2.09	5.25	8.22E-06	1.68E-03
external encapsulating structure	11	2.1	5.24	8.35E-06	1.55E-03
extracellular exosome	19	7.65	2.48	1.52E-04	1.94E-02
extracellular vesicle	19	7.73	2.46	1.75E-04	2.10E-02
extracellular membrane-bounded organelle	19	7.74	2.46	1.76E-04	1.89E-02
extracellular organelle	19	7.74	2.46	1.76E-04	2.00E-02

Panther Protein Class	Observed	Expected	Fold Enrichment	Raw P Value	FDR
protease inhibitor	6	0.48	12.57	1.05E-05	2.06E-03

Reactome Pathways	Observed	Expected	Fold Enrichment	Raw P Value	FDR
LDL remodeling	2	0.02	91.51	3.59E-04	2.98E-02
Plasma lipoprotein remodeling	3	0.12	25.74	2.76E-04	2.38E-02
Plasma lipoprotein assembly, remodeling, and clearance	4	0.24	16.39	1.29E-04	1.29E-02
GRB2:SOS provides linkage to MAPK signaling for Integrins	4	0.05	78.43	4.71E-07	2.35E-04

Integrin signaling	4	0.09	42.23	4.08E-06	1.02E-03
Platelet Aggregation (Plug Formation)	4	0.14	28.90	1.61E-05	3.35E-03
Platelet activation, signaling and aggregation	9	0.95	9.50	5.57E-07	1.98E-04
p130Cas linkage to MAPK signaling for integrins	4	0.05	73.21	5.95E-07	1.85E-04
Regulation of TLR by endogenous ligand	4	0.08	52.29	1.91E-06	5.29E-04
MyD88 deficiency (TLR2/4)	3	0.06	48.44	5.01E-05	5.94E-03
Diseases associated with the TLR signaling cascade	3	0.11	26.57	2.53E-04	2.34E-02
Diseases of Immune System	3	0.11	26.57	2.53E-04	2.43E-02
IRAK4 deficiency (TLR2/4)	3	0.07	45.75	5.83E-05	6.60E-03
Common Pathway of Fibrin Clot Formation	3	0.08	37.43	9.97E-05	1.08E-02
Formation of Fibrin Clot (Clotting Cascade)	5	0.14	35.19	5.35E-07	2.22E-04
Signaling by high-kinase activity BRAF mutants	4	0.13	31.37	1.20E-05	2.71E-03
Oncogenic MAPK signaling	4	0.3	13.39	2.71E-04	2.42E-02
MAP2K and MAPK activation	4	0.14	28.16	1.77E-05	3.40E-03
Signaling by RAF1 mutants	4	0.15	27.45	1.95E-05	3.23E-03
Post-translational protein phosphorylation	10	0.39	25.66	1.39E-11	3.47E-08
Signaling downstream of RAS mutants	4	0.16	24.4	3.00E-05	4.67E-03
Signaling by RAS mutants	4	0.16	24.4	3.00E-05	4.15E-03
Paradoxical activation of RAF signaling by kinase inactive BRAF	4	0.16	24.4	3.00E-05	4.39E-03
Signaling by moderate kinase activity BRAF mutants	4	0.16	24.4	3.00E-05	3.93E-03
Regulation of Insulin-like Growth Factor (IGF) transport and uptake by Insulin-like Growth Factor Binding Proteins (IGFBPs)	10	0.45	22.14	5.43E-11	6.76E-08
Platelet degranulation	9	0.46	19.45	1.54E-09	1.28E-06
Response to elevated platelet cytosolic Ca2+	9	0.48	18.72	2.12E-09	1.32E-06
Signaling by BRAF and RAF1 fusions	4	0.23	17.16	1.09E-04	1.14E-02
Integrin cell surface interactions	5	0.31	16.34	1.80E-05	3.21E-03
Binding and Uptake of Ligands by Scavenger Receptors	4	0.37	10.77	6.01E-04	4.83E-02

Supplemental Table 3. F_{ST} Values across ΔEP spectrum of values.

Mean F_{ST} rank value for UK10K SNPs in ΔEP bins for three population contrasts. Values are for SNPs that are in the UK10K sample and occur with at least 10 derived alleles in the pooled populations of the contrast. For each ΔEP SNP the observed F_{ST} was ranked against that for control alleles of the same derived allele frequency.

ΔEP	Africa vs Eurasia	Europe vs Asia	Great Britain vs Italy
-0.95	0.5615	0.5254	0.4913
-0.85	0.5416	0.5249	0.5097
-0.75	0.5388	0.5219	0.5112
-0.65	0.521	0.5344	0.5106
-0.55	0.5209	0.5211	0.5003
-0.45	0.514	0.511	0.4985
-0.35	0.4986	0.5182	0.5173
-0.25	0.5178	0.4934	0.4936
-0.15	0.5085	0.5328	0.4928
-0.05	0.511	0.5223	0.4485
0.05	0.5076	0.5423	0.5211
0.15	0.5052	0.5544	0.4942
0.25	0.479	0.5825	0.5861
0.35	0.4844	0.532	0.5286
0.45	0.4902	0.547	0.4228
0.55	0.5889	0.5955	0.4614
0.65	0.5295	0.4961	0.4488
0.75	0.5159	0.6301	0.5388
0.85	0.4205	0.4204	0.4972
0.95	0.5693	0.4416	0.5675

Supplementary Table 4. ΔEP measures for fixed and polymorphic alleles

Based on maximum-likelihood rooting estimates of ancestral alleles (see Figure 1 for values based on Ensembl rooting). 95% confidence intervals on the mean, determined by bias-corrected bootstrap, are given in parentheses.

	Negative ΔEP		Positive ΔEP	
Measure	Fixed (%)	Polymorphic (%)	Fixed (%)	Polymorphic (%)
Count	29348 (13.0%)	195747 (87.0%)	6941 (60.2%)	4585 (39.8%)
Mean frequency	1	0.025	1	0.096
Mean Ancestral EP	0.714	0.848	0.122	0.249
Mean Derived EP	0.052	0.017	0.602	0.515
Mean ΔEP	-0.663	-0.832	0.480	0.266

Supplementary Table 5. Statistical power for detecting excess heterozygosity

Selection Coefficient	Probability of rejecting null hypothesis at false positive rate of 0.05
0.0	0.0485
0.0001	0.0545
0.0002	0.0560
0.0005	0.0615
0.001	0.0830
0.002	0.136
0.005	0.417
0.01	0.870
0.02	1.0
0.05	1.0
0.1	1.0

Supplementary Figure 1. Distributions of derived polymorphism frequency in UK10K.

Distribution of derived allele frequency for each ΔEP bin from –1 to +1 in 0.1 increments. Derived allele frequency ranges from singletons (1 copy of the derived allele) to 7241 copies (only one copy of the ancestral allele). The majority of sites are found at low frequencies across all bins.

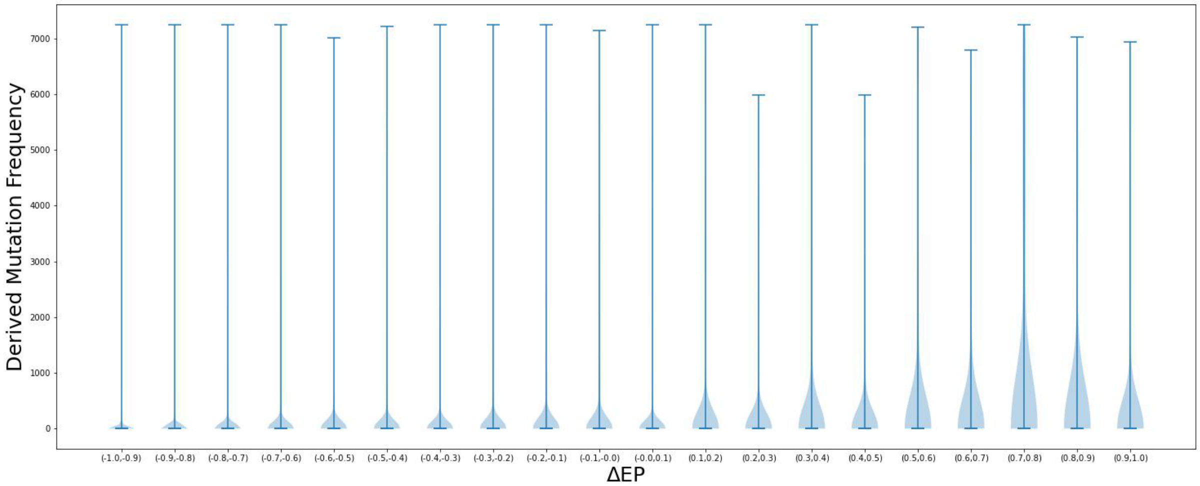

Supplement: Supplement 1 — Supplementary Table 1. Results of simulation-based tests of dispersion of positive ΔEP SNPs. Supplemental Table 2. Gene ontology results Supplemental Table 3. FST Values across ΔEP spectrum of values. Mean FST rank value for UK10K SNPs in ΔEP bins for three population contrasts. Values are for SNPs that are in the UK10K sample and occur with at least 10 derived alleles in the pooled populations of the contrast. For each ΔEP SNP the observed FST was ranked against that for control alleles of the same derived allele frequency. Supplementary Table 4. ΔEP measures for fixed and polymorphic alleles. Based on maximum-likelihood rooting estimates of ancestral alleles (see Figure 1 for values based on Ensembl rooting). Simulated mean ΔEP was calculated for each SNP by considering all possible non-synonymous mutations and the corresponding EP value for the resulting amino acid in proportion to their mutation probabilities based on empirical estimates. 95% confidence intervals on the mean, determined by bias-corrected bootstrap, are given in parentheses. Supplementary Table 5. Statistical power for detecting excess heterozygosity. Supplementary Figure 1. Distributions of derived polymorphism frequency in UK10K. Distribution of derived allele frequency for each ΔEP bin from −1 to +1 in 0.1 increments. Derived allele frequency ranges from singletons (1 copy of the derived allele) to 7241 copies (only one copy of the ancestral allele). The majority of sites are found at low frequencies across all bins. [file media-1.pdf]
